# Isolated serosal liver infiltration mimicking cirrhosis in gastroesophageal junction adenocarcinoma: A report of 2 cases

**DOI:** 10.1016/j.radcr.2025.10.043

**Published:** 2025-11-09

**Authors:** Jaya Gounder, Adam Frankel, Stanley Ngai

**Affiliations:** a Department of Radiology, Princess Alexandra Hospital, 199 Ipswich Road, Woolloongabba, 4102, Queensland, Australia; b Griffith University, 1 Parklands Drive, Southport, 4215, Queensland, Australia; c Department of Surgery, Princess Alexandra Hospital, 199 Ipswich Road, Woolloongabba, 4102, Queensland, Australia; d Faculty of Medicine, University of Queensland, 11 Wyndham Street, Herston, 4006, Queensland, Australia

**Keywords:** Serosal infiltration, Pseudocirrhosis, Peritoneal neoplasms, Esophageal junction

## Abstract

Pseudocirrhosis is a rare radiological phenomenon characterized by a cirrhosis-like morphology of the liver caused by metastatic disease without underlying chronic liver disease. We present 2 such cases. The first is a 79-year-old male with newly diagnosed gastro-esophageal junction (GOJ) adenocarcinoma with serosal infiltration of the hepatic capsule without peritoneal carcinomatosis. The second case is a 77-year-old female with GOJ adenocarcinoma showing radiological features of peritoneal infiltration and pseudocirrhosis. Neither patient had any history of chronic liver disease. These cases highlight the importance of recognizing pseudocirrhosis, particularly when imaging findings are discordant with clinical or laboratory data, as the diagnosis significantly alters staging and management.

## Introduction

Gastro-esophageal junction (GOJ) adenocarcinoma is an aggressive malignancy often presenting with local invasion and/or distant metastases due to its insidious onset [1]. Staging typically involves computed tomography (CT) and positron emission tomography (PET), with common metastatic sites include lymph nodes, peritoneum, and solid organs (liver, lung and bone) [2]. Diagnosis of peritoneal metastasis is important as it is usually incurable [2]. Alcohol abuse is a risk factor for both gastroesophageal malignancies and chronic liver disease [3]. Cirrhosis, pseudocirrhosis and cirrhosis-like appearances of the liver as sequalae of peritoneal disease all have important diagnostic and treatment implications [4].

The diagnosis of pseudocirrhosis in the setting of malignancy remains challenging, as it mimics cirrhosis both clinically and radiologically, including secondary findings such as portal hypertension [5]. The radiological features of cirrhosis and pseudocirrhosis include capsular retraction, caudate lobe hypertrophy and parenchymal atrophy [6]. While CT and MRI can detect morphological surface changes within the liver, PET imaging may fail to demonstrate metabolic activity in serosal infiltrative disease. Histological confirmation remains essential for equivocal cases.

This case report highlights the importance of considering pseudocirrhosis in the differential diagnosis of cirrhotic morphology in patients with GOJ adenocarcinoma, particularly when clinical and laboratory findings are discordant with imaging.

## Case presentation

### Case 1

**Fig. 1:**
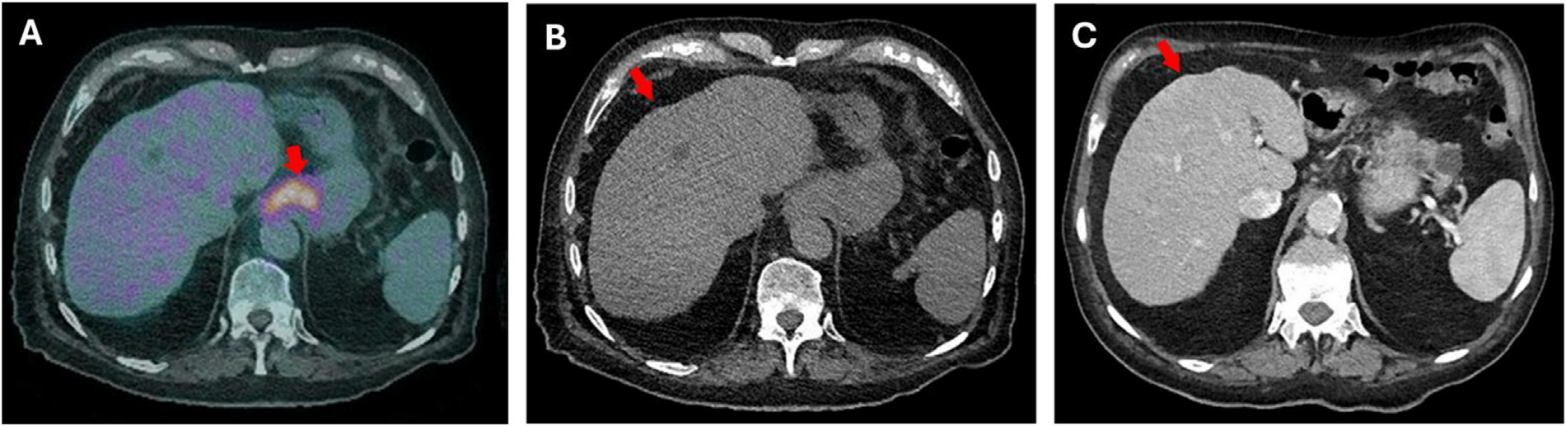
(A) Axial 18F-FDG PET image at the level of the gastroesophageal junction (GOJ) demonstrates intense FDG avidity corresponding to the primary GOJ malignancy as indicated by the red arrow. (B) Axial low-dose noncontrast CT (from PET/CT) demonstrates nodular hepatic contours without discrete focal hepatic lesions indicated by the red arrows and (C) Axial postcontrast CT of the liver shows nodular liver margins indicated by the red arrows, with no evidence of focal parenchymal lesions.

A 79-year-old male presented with 12 months of progressive dysphagia and a 20 kg weight loss. He had no previous medical or family history of cirrhosis, and his examination was unremarkable on presentation. Upper endoscopy revealed a near-obstructing distal oesophageal lesion. Biopsies confirmed moderately differentiated adenocarcinoma of the GOJ. Staging FDG PET demonstrated intense uptake at the GOJ and gastric cardia, with no evidence of nodal or distant metastases (Fig. 1A). However, CT revealed nodular hepatic contour suggestive of cirrhosis despite no prior liver disease (Fig. 1B and C). Liver function tests showed chronically elevated gamma-glutamyl transferase (GGT) and ferritin levels.

**Fig. 2:**
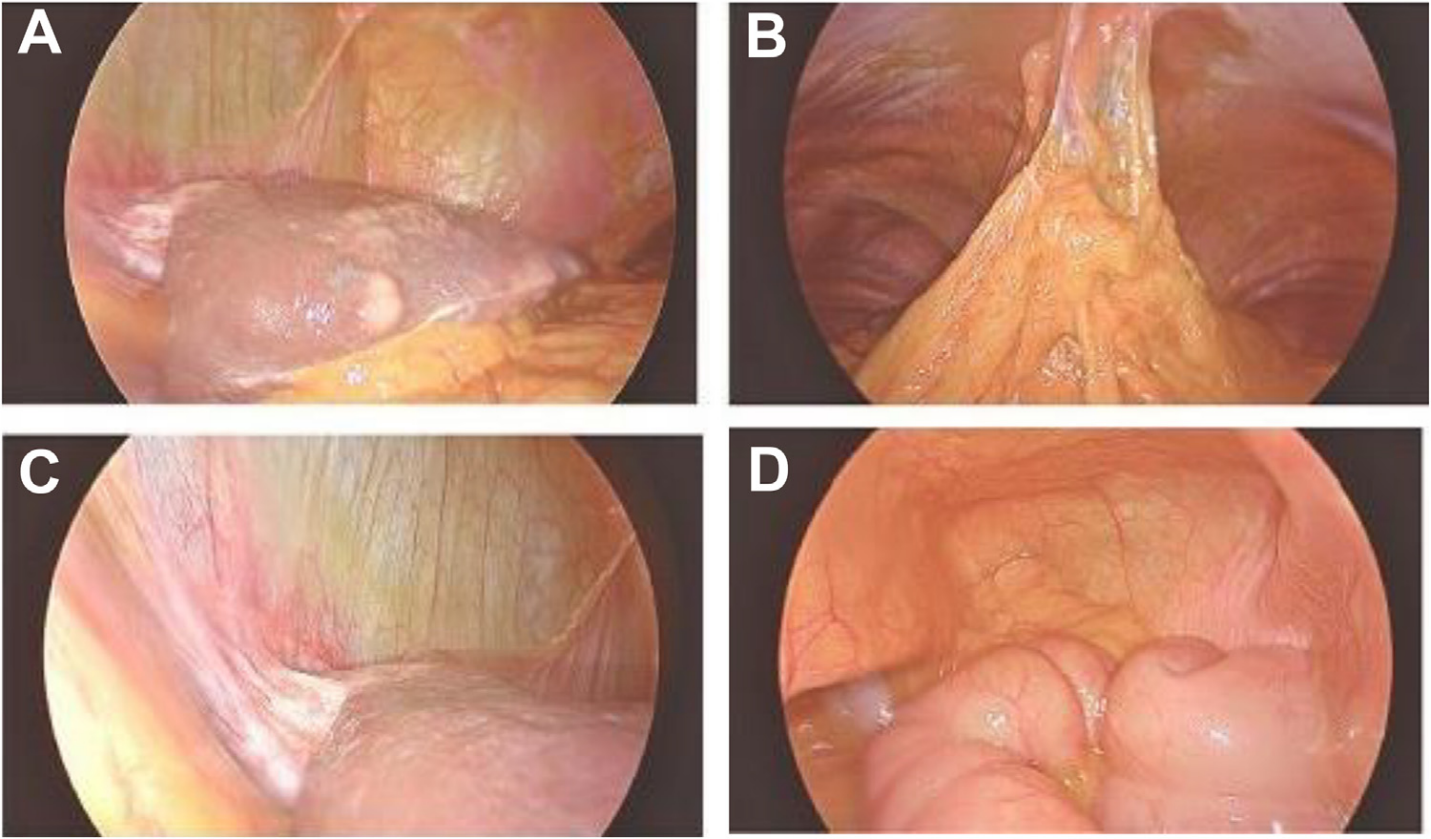
Intraoperative laparoscopic images from a 79-year-old male with gastroesophageal junction adenocarcinoma. Panel (A) show the nodular, cirrhosis-like surface of the liver consistent with serosal tumor infiltration. Panels (B) and (D) show the omentum without evidence of tumor involvement and Panel (C) demonstrate the peritoneal surfaces, which appear free of metastatic deposits.

Laparoscopy revealed a nodular liver surface without peritoneal involvement (Fig. 2A–D). Peritoneal washings were negative for malignancy. Targeted liver biopsies identified adenocarcinoma infiltration confined to the hepatic serosa (visceral peritoneum), with preserved parenchyma and no fibrosis. The disease was reclassified as stage IV and the patient was referred for supportive management.

### Case 2

**Fig. 3:**
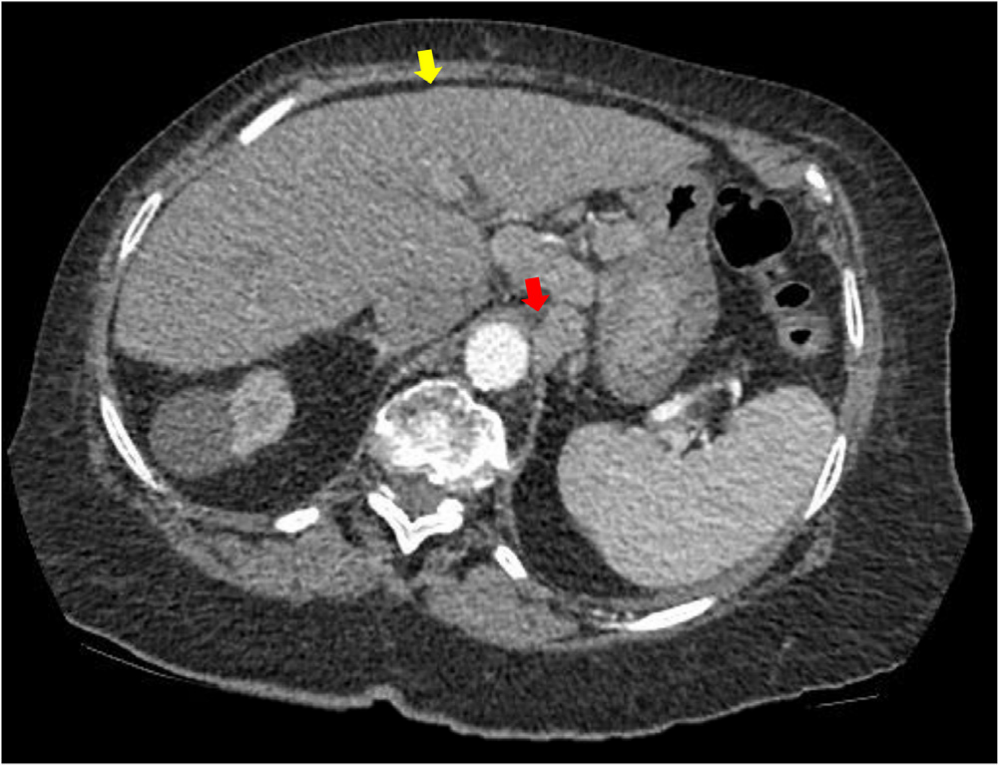
Axial postcontrast CT demonstrating the primary gastroesophageal mass indicated by the red arrow and nodular hepatic contours consistent with a cirrhosis-like appearance indicated by the yellow arrow.

A 77-year-old female with newly diagnosed gastric cardia adenocarcinoma underwent staging CT, which showed irregular liver margins, the GOJ mass, irregular liver margins, abnormal abdominal lymph nodes, omental nodules and ascites (Fig. 3). She had a history of hypertension and chronic kidney disease but no personal or family history of cirrhosis. Her examination on presentation was unremarkable. Laparoscopy confirmed both peritoneal carcinomatosis and serosal infiltration of the liver with no evidence of parenchymal liver metastases. The radiological findings mimicked cirrhosis, but histology confirmed tumor involvement was limited to the liver capsule and peritoneum. Given the extent of metastatic disease, the patient was managed with supportive care.

## Discussion

Pseudocirrhosis is a radiological diagnosis describing the morphological changes of the liver which mimic cirrhosis, however, arise in the setting of metastatic malignancy. The imaging features are characterized by hepatic surface nodularity, lobular atrophy and capsular retraction in the absence of classical histopathological cirrhosis [7]. While most frequently described in patients with metastatic breast cancer, cases have also been reported in lung and gastrointestinal malignancies, including gastric and esophageal cancers [8]. A systematic review by Villani et al. found that most patients clinical signs of portal hypertension (commonly ascites) with a median survival of 2 months [5].

The pathogenesis of pseudocirrhosis is poorly understood. Proposed mechanisms include desmoplastic stromal reactions, capsular retraction from infiltrative disease and nodular regenerative hyperplasia as a possible response to chemotherapy [7]. It may also occur in chemotherapy-naïve patients, suggesting tumor infiltration alone may be sufficient [9].

In GOJ adenocarcinoma, metastases can rarely be confined to the serosa (ie, contained within the Glissonian capsule/visceral peritoneum envelope), mimicking cirrhosis. Most reported cases of pseudocirrhosis in GI malignancies involve either diffuse parenchymal metastases or peritoneal (ie, trans-coelomic) spread [4,10]. Our cases highlight a rare but important scenario in which isolated serosal infiltration of the liver without parenchymal or peritoneal metastases can mimic pseudocirrhosis on imaging. Most literature describes pseudocirrhosis in the context of parenchymal infiltration or widespread peritoneal disease [4,9,10]. The presence of only serosal involvement as in our first case is extremely uncommon and has important implications for staging and management. In contrast, our second case demonstrates the more typical coexistence of serosal and peritoneal disease, although this is also a rare phenomenon.

Accurate diagnosis is essential, as management and prognosis differ significantly between pseudocirrhosis, isolated serosal disease and classic cirrhosis. The shared risk factor of alcohol abuse is a confounder in interpretation. Direct visualization and histopathological confirmation are usually required, particularly if clinical features and imaging are discordant [2].

## Conclusion

Isolated serosal infiltration of the liver by gastro-esophageal malignancy is a rare but important mimic of pseudocirrhosis. Differentiating this pattern from diffuse parenchymal, peritoneal metastatic disease or cirrhosis is essential to guide staging and management. Clinicians and radiologists should be aware of this phenomenon in patients with new-onset cirrhotic liver morphology and have a low threshold for laparoscopy and histopathological confirmation.

## Patient consent

Complete written informed consent was obtained from the patient for the publication of this study and accompanying images.
